# Phylogenetic Relationships within the Nematode Subfamily *Phascolostrongylinae* (Nematoda: *Strongyloidea*) from Australian Macropodid and Vombatid Marsupials

**DOI:** 10.3390/microorganisms9010009

**Published:** 2020-12-22

**Authors:** Tanapan Sukee, Ian Beveridge, Ahmad Jawad Sabir, Abdul Jabbar

**Affiliations:** Department of Veterinary Biosciences, Melbourne Veterinary School, Faculty of Veterinary and Agricultural Sciences, The University of Melbourne, Werribee, VIC 3030, Australia; ibeve@unimelb.edu.au (I.B.); ahmad.sabir@unimelb.edu.au (A.J.S.)

**Keywords:** Phascolostrongylinae, Strongyloidea, parasites, macropods, wombats, internal transcribed spacers, ribosomal DNA, phylogenetics

## Abstract

The strongyloid nematode subfamily Phascolostrongylinae comprises parasites of the large intestine and stomach of Australian macropods and wombats. In this study, we tested the phylogenetic relationships among the genera of the Phascolostrongylinae using the first and second internal transcribed spacers of the nuclear ribosomal DNA. Monophyly was encountered in the tribe Phascolostrongylinea comprising two genera, *Phascolostrongylus* and *Oesophagostomoides*, found exclusively in the large intestine of wombats. The tribe Hypodontinea, represented by the genera *Hypodontus* and *Macropicola* from the ileum and large intestine of macropods, was also found to be monophyletic. The tribe Macropostrongyloidinea, comprising the genera *Macropostrongyloides* and *Paramacropostrongylus*, was paraphyletic with the species occurring in the stomach grouping separately from those found in the large intestines of their hosts. However, *Macropostrongyloides* *dissimilis* from the stomach of the swamp wallaby and *Paramacropostrongylus toraliformis* from the large intestine of the eastern grey kangaroo were distinct from their respective congeners. This study provided strong support for the generic composition of the tribe Phascolostrongylinea. The unexpected finding of *M. dissimilis* and *P. toraliformis* being distantly related to their respective congeners suggests a requirement for future taxonomic revision that may warrant separation of these species at the generic level.

## 1. Introduction

Australian macropodid (Family Macropodidae) and vombatid (Family Vombatidae) marsupials are parasitised by a diverse array of strongyloid nematodes that are classified in the subfamilies Cloacininae and Phascolostrongylinae [1]. The subfamily Cloacininae is found exclusively in the oesophagus and stomach of macropods (kangaroos and wallabies) [2]. This subfamily has been the focus of previous morphological and molecular studies due to their extensive diversity, high prevalence and large burden of nematodes present in the hosts [2]. Conversely, fewer studies have been conducted on the subfamily Phascolostrongylinae, mostly due to the significantly smaller number of species, occurring in low abundance and often encountered sporadically [2]. The subfamily Phascolostrongylinae is currently composed of seven genera found in macropodid and vombatid marsupials. Some of the genera possess unusual morphological features, which may have contributed to difficulties in previous taxonomic revisions [3,4].

The first phylogenetic classification of the superfamily Strongyloidea was based on morphological characters in which emphasis on the female ovejector followed by the male copulatory bursa and buccal capsules placed the nematodes according to host groups [3,4]. However, this classification led to the hypothesis that the strongyloid nematodes of Australian marsupials were of polyphyletic origins. The subfamily Phascolostrongylinae was initially characterised by a Y-shaped ovejector and two branches of the dorsal ray of the male bursa and comprised four genera found in the intestines of macropodid and vombatid marsupials. Although they shared identical ovejector and bursal features as the Phascolostrongylinae, the intestinal parasites of kangaroos and wallabies, the genera *Hypodontus* and *Macropicola* were placed in the subfamily Strongylinae with other nematodes from horses and elephants due to their uniquely large and globular buccal capsules [3]. *Corollostrongylus*, exclusive to the intestine of the musky rat-kangaroo, *Hyspiprymnodon moschatus*, also possesses a globular buccal capsule. However, because of its J-shaped ovejector, this genus was placed in the subfamily Chabertiinae alongside the nematodes of rodents and domestic ruminants [3].

Subsequently, an alternative classification system was proposed for strongyloid nematodes of Australian marsupials, based on the argument that greater emphasis on the male reproductive features would result in the monophyly of this group [1]. Consequently, the genera *Hypodontus*, *Macropicola* and *Corollostrongylus* were added to the subfamily Phascolostrongylinae and it was further subdivided into three tribes [1]. One tribe, Phascolostrongylinea, comprised *Phascolostrongylus turleyi* and four species of *Oesophagostomoides*, all occurring within the colon of wombats. Another tribe, Macropostrongylinea, consisting of the genera *Macropostrongyloides* and *Paramacropostrongylus*, is found in the stomach and large intestines of macropodid hosts. Finally, the tribe Hypodontinea, from large intestines of macropods, comprised *Hypodontus*, *Macropicola* and *Corollostrongylus* [1].

Following Beveridge’s [1] reclassification, several molecular studies have utilised allozyme and DNA sequencing data to detect genetic variation within the genera *Hypodontus* [5,6,7], *Paramacropostrongylus* [8,9], and *Macropostrongyloides* [10,11]. However, phylogenetic studies at the subfamily level have been neglected. One study attempted to examine the relationships within the Phascolostrongylinae based on the second internal transcribed spacer (ITS-2) subunit of the nuclear ribosomal DNA data [12]. This technique provided an opportunity to address the gap in research of the strongyloid of Australian marsupials. However, the findings were inconclusive due to the limited number of species analysed within the subfamily Phascolostrongylinae and the analysis of only one internal transcribed spacer [12]. Other molecular studies have included both the sequences of the first and second internal transcribed spacers (ITS-1 and ITS-2, respectively [ITS+]) and have found these markers to be extremely useful for assessing phylogenetic relationships among closely related taxa of strongyloid nematodes in Australian marsupials [6,7,8,9,11,13,14,15,16]. Although the relationships between the tribes within the subfamily *Phascolostrongylinae* proposed by Beveridge [1] still remain untested, analyses of the ITS markers could provide molecular support for Beveridge’s [1] morphological classification.

The current study characterised the ITS+ sequences of five genera within the Phascolostrongylinae (i.e., *Paramacropostrongylus*, *Hypodontus*, *Macropicola*, *Oesophagostomoides* and *Phascolostrongylus*). Following comparative analyses of the current ITS+ sequence data with published sequences of *Macropostrongyloides* spp., phylogenetic relationships within the Phascolostrongylinae were determined.

## 2. Materials and Methods

### 2.1. Collection of Specimens

Adult nematodes of *Paramacropostrongylus* (*P.*) *toraliformis* (*n* = 18), *P. typicus* (*n* = 11), *P. iugalis* (*n* = 17), *Phascolostrongylus* (*Pa.*) *turleyi* (*n* = 48), *Oesophagostomoides* (*O.*) *longispicularis* (*n* = 57), *O. stirtoni* (*n* = 14), *O. giltneri* (*n* = 3) and *Macropicola* (*M.*) *ocydromi* (*n* = 3) were collected from road-killed or commercially culled hosts and stored at −80 °C in the frozen parasite collection at the Veterinary School of the University of Melbourne.

Specimens were collected under the following state-issued permits: Victorian Department of Sustainability and Environment 90-053, 93-016, 10000434, 100003649; Queensland Department of Environment and Heritage Protection WA 00006125.

### 2.2. Morphological Identification of Nematodes

Upon thawing, the nematodes were dissected, the anterior and posterior extremities were cleared in lactophenol and examined using an Olympus BH-2 microscope. The mid-sections of worms were processed for molecular studies. The anterior and posterior extremities of specimens used for morphological studies were then stored in 70% ethanol and deposited in the Australian Helminthological Collection (AHC) of the South Australian Museum, Adelaide (SAM) (Table 1). Host nomenclature follows Jackson and Grooves [17].

### 2.3. Molecular Characterisation of Nematodes

Genomic DNA (gDNA) was isolated from the mid-sections of nematodes using a small-scale sodium-dodecyl-sulphate/proteinase K extraction procedure [18] followed by purification using either a mini-column (Wizard™ Clean-Up, Promega, Madison, WI, USA) for *Paramacropostrongylus* or the QIamp DNA Micro Kit (Qiagen, Germany) for all other worms following manufacturers’ protocols. The concentration and purity of each DNA sample were determined spectrophotometrically (ND-1000 UV-VIS spectrophotometer v.3.2.1; NanoDrop Technologies, Inc., Wilmington, DE, USA).

The ITS-1, 5.8S and ITS-2 regions (ITS+) within the rDNA were amplified by Polymerase Chain Reaction (PCR) using the primers NC16 (5′-AGTTCAATCGCAATGGCTT-3′) and NC2 (5′-TTAGTTTCTTTTCCTCCGCT-3′) [19]. Each PCR was conducted in 50 μL volume containing 2 μL of DNA template, 10 mM of Tris-HCl (pH 8.4), 50 mM of KCl (Promega), 3.5 mM of MgCl2, 250 μM of deoxynucleotide triphosphate (dNTP), 100 pmol of each primer and 1 U of GoTaq polymerase (Promega). The PCR conditions used were: 94 °C for 5 min, then 35 cycles of 94 °C for 30 s, 55 °C for 20 s and 72 °C for 20 s, followed by 72 °C for 5 min. Negative (no DNA template) and positive controls (*Labiosimplex bipapillosus* and *Haemonchus contortus* gDNA) were included in the PCR analyses. An aliquot (5 μL) of each amplicon was subjected to agarose gel electrophoresis. Gels (1.5% gels in 0.5 TAE buffer containing 20 mM Tris, 10 mM acetic acid, 0.5 mM EDTA) were stained using GelRed Nucleic Acid Gel Stain (Biotium GelRed stain, Fisher Scientific, Waltham, MA, USA) and photographed using a gel documenting system (Kodak Gel Logic 1500 Imaging System, Eastman Kodak Company, Rochester, NY, USA).

Amplicons were purified using shrimp alkaline phosphate and exonuclease I [20] before automated Sanger DNA sequencing using a 96-capillary 3730xl DNA Analyser (Applied Biosystems, Foster City, CA, USA) at Macrogen, Inc., Seoul, Korea. The ITS+ was sequenced using the primers NC16 and NC2 in separate reactions. The quality of the sequences was assessed in the Geneious R10 software (Biomatters Ltd., Auckland, New Zealand; www.geneious.com). Polymorphic sites were designated using the International Union of Pure and Applied Chemistry (IUPAC) codes. DNA sequences have been submitted to the GenBank database under the accession numbers MT396193-MT396208 (Table 1). Published ITS-1 and ITS-2 sequences of *Macropostrongyloides* spp. were obtained from GenBank under accession numbers MK842122-MK842146. The ITS-2 sequences of *Hypodontus macropi* were also acquired from GenBank (HE866717 and HE866724); however, the ITS-1 sequences were from unpublished data. *Hypodontus macropi* is a species complex comprising at least 10 genotypes based on the ITS-2 sequence data [7], and only two representative genotypes of *H. macropi* were included in the tree.

### 2.4. Phylogenetic Analyses

The ITS sequences were aligned using the log-expectation (MUSCLE) algorithm in the software MEGA 7.0.26 [21]. Pairwise comparisons among sequences were determined using Geneious Prime 2019.2.1 [22]. Phylogenetic relationship among the ITS+ sequences was estimated using the distance-based Neighbour-joining (NJ) algorithm in MEGA and the unconstrained branch length Bayesian inference (BI) analysis in MrBayes [23]. The NJ analyses were conducted based on the number of differences as evolutionary distances [24], including transitions and transversions among nematode species. Rates among sites were considered uniform, and gaps were treated using pairwise deletion with 10,000 bootstrap replicates and are reported as bootstrap (bs) values [25]. The most appropriate partition scheme and the evolutionary model for the BI analysis were determined using PartitionFinder V. 2.0 [26] under the Akaike’s Information Criterion. The data were partitioned into subset 1 (ITS-1) subset 1 and subset 2 (ITS-2). The evolutionary model assigned for both data subsets was nst = 6 with a proportion of invariable sites. The BI analysis was conducted in MrBayes with the Markov chain Monte Carlo with three heated and one cold chain for 2 million generations sampled every 1000th generations for three runs to ensure convergence and calculate posterior probabilities (pp). At the end of each run, the standard deviation of split frequencies was <0.01, and the Potential Scale Reduction Factor equalled one. For each analysis, a 50% majority rule consensus tree was constructed based on the final 75% of trees. The ITS+ sequence of *Cloacina cadmus* from the quokka, *Setonix brachyuris* (GenBank accession no. MF284677.1), from the related subfamily Cloacininae was used as the outgroup. The topology of trees was visualised using the software FigTree v1.4.4 (http://tree.bio.ed.ac.uk/software/figtree/).

## 3. Results

### Molecular Characterisation of Nematodes

Amplicons of the ITS-1, the interspacing 5.8S gene, and ITS-2 generated were approximately 1000 bp. Subsequent to quality trimming and sequence alignment, two unique sequences were generated each for *P. toraliformis*, *P. typicus*, *P. iugalis*, *Ph. turleyi*, *O. giltneri* and *O. stirtoni*, and one for *O. longispicularis* and *M. ocydromi* (Table 1).

The ITS-1 and ITS-2 sequences ranged from 370 to 406 bp and 217 to 292 bp, respectively. The GC content ranged from 39.80 to 46.10% and 38.00 to 41.90% for the ITS-1 and ITS-2 sequences, respectively (Table 2). The 5.8S gene contained 153 bp for all species sequenced, consistent with other species of strongyloid nematodes in Australian marsupials.

The concatenated ITS-1 and ITS-2 (ITS+) sequence variation among different species ranged from 0.20–34% (Table 3). The two most distant sequences (34% sequence difference) were those of *H. macropi* (G12) and *O. stirtoni* (41W2). The genus *Macropostrongyloides* exhibited the highest intrageneric variation (3–22.8%), with *Ma. woodi* and *Ma. dissimilis* being the most distantly related species within the genus. In contrast, *Oesophagostomoides* from the wombat displayed the least genetic variation (0.5–2.9%) (Table 3).

**Figure 1:**
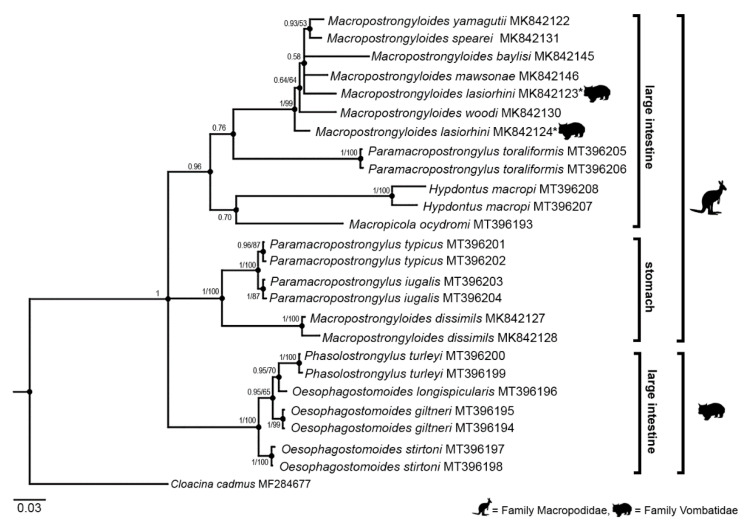
Phylogenetic analysis of the ITS+ rDNA sequences the *Phascolostrongylinae* species from macropodid (kangaroo icon) and vombatid (wombat icon) hosts. The sequence data were analysed using the Bayesian Inference (BI) and Neighbour-Joining (NJ) methods. Nodal support is given as a posterior probability followed by bootstrap value for BI and NJ, respectively. *Cloacina cadmus* from the quokka, *Setonix brachyurus* (GenBank accession no. MF284677), was used as the outgroup. The scalebar indicates the number of inferred substitutions per nucleotide site.

The length of the MUSCLE alignment was 816 bp (448 and 368 bp in ITS-1 and ITS-2, respectively). The alignment consisted of 407 conserved sites, 336 variable sites and 211 parsimoniously informative sites (Supplementary File 1; Figure S1). The phylogenetic trees derived from the ITS+ sequences generated very similar tree topologies for the BI and NJ analyses; therefore, only the BI tree is presented in Figure 1. The phylogenetic reconstruction showed that all species within the Phascolostrongylinae formed a monophyletic group with strong nodal support (posterior probability (pp) = 1) (Figure 1). The tree topology showed three major clades. The first clade contained the genera *Phascolostrongylus* and *Oesophagostomoides* from vombatid hosts (pp = 1, bootstrap support (bs) = 100), the second clade comprised the genera *Paramacropostrongylus* and *Ma. dissimilis* (pp = 1, bs = 100) and the last clade consisted of *Hypodontus*, *Macropicola*, *P. toraliformis* and the remaining species of *Macropostrongyloides* (pp = 0.96). The first clade containing genera occurring specifically in wombats was subdivided based on the host species in which they occurred. *Oesophagostomoides stirtoni* from *L. latifrons* formed a separate subclade to the remaining species that occur in *V. ursinus*. In the second clade, both ITS+ sequences of *Ma. dissimilis* occurred externally to the remaining species within the genus and were instead clustered as a sister clade to *P. typicus* and *P. iugalis* with strong branch support (pp = 1, bs = 100). The third and largest clade was further subdivided into two clades. The genera *Hypodontus* and *Macropicola* were sisters in one clade, whilst *P. toraliformis* occurred as sister to the clade containing the majority of the species of *Macropostrongyloides*. However, both clades had low nodal support (pp = 0.70 and pp = 0.76, respectively).

## 4. Discussion

The current study examined the phylogenetic relationships of 17 morphospecies within the Phascolostrongylinae from Australian marsupials based on the ITS+ sequences. The phylogenetic relationships inferred from the ITS+ data partially supported the current morphological classification of Beveridge [1]. It was found that the genera *Phascolostrongylus*, *Oesophagostomoides*, *Paramacropostrongylus*, *Macropostrongyloides*, *Hypodontus* and *Macropicola* were monophyletic. However, the tribe Macropostrongyloidinea was paraphyletic, contrary to the morphological findings of Beveridge [1].

The ITS+ sequence data were concordant with the inclusion of the genera *Hypodontus* and *Macropicola* within the Phascolostrongylinae as proposed by Beveridge [1]. Although not strongly supported, the genera *Hypodontus* and *Macropicola* formed a clade, consistent with the tribe Hypodontinea of Beveridge [1]. Lichtenfels [3] initially placed these genera within the subfamily Strongylinae based on morphological characters. However, Beveridge [1] subsequently transferred the Phascolostrongylinae to the family Charbertiidae in addition to placing *Hypodontus* and *Macropicola* within the Phascolostrongylinae based on dorsal ray and ovejector types. The ITS+ sequences of *Corollostrongylus* from the large intestine of the musky rat-kangaroo, *Hypsiprymnodon moschatus* [27] were not included in the current study due to the unavailability of material for molecular analysis. This genus was originally placed in the Chabertiinae by Lichtenfels [3] and was moved to the tribe Hypodontinea within the Phascolostrongylinae by Beveridge [1]. To further resolve the relationships within the Phascolostrongylinae, additional studies are required with the inclusion of the *Corollostrongylus*.

This study supported the classification of the genera *Phascolostrongylus* and *Oesophagostomoides* in the tribe Phascolostrongylinea, parasitic in the colon of vombatid hosts [1]. Based on the current phylogenetic tree, these two genera share a common ancestor with the genera of the Phascolostrongyline from macropodid marsupials. This is consistent with the hypothesis that *Phascolostrongylus* and *Oesophagostomoides* may have arisen by host-switching and evolved in parallel with species parasitic in macropodid hosts [1]. *Oesophagostomoides* and *Phascolostrongylus*, in addition to *Ma. lasiorhini*, are presently the only strongyloids genera known to infect wombats. The grouping of *Ma. lasiorhini* with the other species of *Macropostrongyloides* from macropodid marsupials suggests that this species may have also evolved by means of host-switching from macropodid marsupials [1]. However, one species excluded from the analyses due to lack of available material was *Oesophagostomoides eppingensis* from the colon of the critically endangered northern hairy-nosed wombat, *Lasiorhinus krefftii* [28].

Finally, contrary to Beveridge’s morphological findings [1], these data support the paraphyly of the tribe Macropostrongyloidinea. The genera *Macropostrongyloides* and *Paramacropostrongylus* were split between two clades, implying paraphyly. Instead of grouping within the clade containing the majority of the species of *Macropostrongyloides*, *Ma. dissimilis* formed a strongly supported association with *P. typicus* and *P. iugalis*. This relationship may be related to the predilection site within the hosts. These three species are currently the only strongyloid nematodes, apart from the subfamily Cloacininae, known to occur in the stomachs of their hosts. Although *P. toraliformis* occurs in the large intestines of its host, as do most *Macropostrongyloides* spp., the position of *P. toraliformis* as a sister taxon to the *Macropostrongyloides* clade lacked strong statistical support. Further research is required to better understand its relationship. Additionally, the grouping of *Ma. dissimilis*, *P. typicus* and *P. iugalis* is in concordance with previous morphological hypotheses [1] in that these species possess both features of Type-II (or J-shaped) ovejectors and Type-I (or Y-shaped) ovejectors. These features suggest that *Ma. dissimilis*, *P. typicus* and *P. iugalis* may represent an intermediate link in the evolution of the Phascolostrongylinae and the Cloacininae [1]. However, this assumption requires additional evidence from both molecular and morphological studies on a wider range of species including the subfamily Cloacininae.

The similarities between the sequences of *P. iugalis* and *P. typicus* (0.6–0.8% sequence difference) from the current study were consistent with previous electrophoretic data [9]. However, the electrophoretic data showed evidence of hybridisation between *P. iugalis* and *P. typicus* in regions of New South Wales in which the two grey kangaroo host species, *Macropus giganteus* and *Macropus fuliginosus*, occur in sympatry [9]. The current study included specimens from both hosts in the region of sympatry between Nyngan and Bourke, New South Wales. However, a comparison of the ITS+ sequences of *P. typicus* and *P. iugalis* did not reveal any evidence of hybridisation between these two species.

## 5. Conclusions

In conclusion, the phylogenetic analyses of the ITS+ sequence data presented herein provided greater insights into the interrelationships within the Phascolostrongylinae. The current molecular data supported the monophyletic grouping of the Phascolostrongylinae consistent with the classification of Beveridge [1]. However, there were some inconsistencies between the phylogenetic relationships and the morphological classification, suggesting the requirement of further taxonomic revision of *M. dissimilis* and *P. toraliformis*. Future molecular studies utilising multiple gene regions or protein sequences [29] may be required to determine the evolutionary processes within the Phascolostrongylinae.

## Figures and Tables

**Table 1:** Species within the subfamily Phascolostrongylinae included in the current study shown with information of the host and localities from which they were collected. The GenBank Accession numbers of the unique ITS + sequences are also included in addition to the SAM number.

Species	Host	Location	Coordinates	Voucher No.	SAM No.	GenBank
*Macropicola ocydromi*	*Macropus fuliginosus*	Waroona, WA	32°57′ S, 115°55′ E	24C1.1	49040	MT396193
*Oesophagostomoides giltneri*	*Vombatus ursinus*	Flowerdale, Vic	37°19′ S, 145°19′ E	41Z1	49022	MT396194
*O. giltneri*	*V. ursinus*	Flowerdale, Vic	37°19′ S, 145°19′ E	41V1	49038	MT396195
*O. giltneri*	*V. ursinus*	Bullengarook, Vic	37°28′ S, 144°21′ E	F23	48995	
*Oesophagostomoides longispicularis*	*V. ursinus*	Licola, Vic	37°39′ S, 146°39′ E	47K.4-8	49034	MT396196
*O. longispicularis*	*V. ursinus*	Ensay, Vic	37°27′ S, 147°49′ E	47E.1-3	49026	
*O. longispicularis*	*V. ursinus*	Ensay, Vic	37°27′ S, 147°49′ E	47F.1, 4	49028	
*O. longispicularis*	*V. ursinus*	Hazelwood, Vic	38°19′ S, 146°24′ E	47B.1, 5, 7	49024	
*O. longispicularis*	*V. ursinus*	Boolarra, Vic	38°24′ S, 146°12′ E	47G.9	49029	
*O. longispicularis*	*V. ursinus*	Mirboo North, Vic	38°22′ S, 146°10′ E	47H.10-18	49031	
*Oesophagostomoides stirtoni*	*Lasiorhinus latifrons*	Swan Reach, SA	34°34′ S, 139°36′ E	41W1.2	49037	MT396197
*O. stirtoni*	*L. latifrons*	Swan Reach, SA	34°34′ S, 139°36′ E	41W1.4	49036	MT396198
*Phascolostrongylus turleyi*	*V. ursinus*	Flowerdale, Vic	37°19′ S, 145°19′ E	42L1	49035	MT396199
*Pa. turleyi*	*V. ursinus*	Delburn, Vic	38°19′ S, 146°17′ E	47A.3	49023	
*Pa. turleyi*	*V. ursinus*	Nowa Nowa, Vic	37°43′ S, 148°04′ E	10Z1	49039	MT396200
*Pa. turleyi*	*V. ursinus*	Boho South, Vic	36°47′ S, 145°47′ E	41Q1.3, 5	49021	
*Pa. turleyi*	*V. ursinus*	Flowerdale, Vic	37°19′ S, 145°19′ E	42L2.1-5	49035	
*Pa. turleyi*	*V. ursinus*	Ensay, Vic	37°27′ S, 147°49′ E	47E5-6, 8	49027	
*Pa. turleyi*	*V. ursinus*	Mirboo North, Vic	38°22′ S, 146°10′ E	47J.7-8	49032	
*Pa. turleyi*	*V. ursinus*	Boolarra, Vic	38°24′ S, 146°12′ E	47G12-14	49030	
*Pa. turleyi*	*V. ursinus*	Fish Creek, Vic	38°74′ S, 146°70′ E	47C1-3	49025	
*Paramacropostrongylus typicus*	*Macropus giganteus*	65 km NW of Nyngan, NSW	31°17′ S, 147°15′ E	14B28	36783	MT396201
*P. typicus*	*M. fuliginosus*	Menzies, WA	29°49′ S, 121°05′ E	36D2	45534	
*P. typicus*	*M. fuliginosus*	Menzies, WA	29°49′ S, 121°05′ E	36A1	45534	
*P. typicus*	*M. fuliginosus*	163 km NW of Nyngan, NSW	30°10′ S, 146°52′ E	14C14	36786	
*P. typicus*	*M. giganteus*	Girilambone, NSW	31°06′ S, 147°04′ E	14R1	36787	
*P. typicus*	*M. fuliginosus*	65 km NW of Nyngan, NSW	31°17′ S, 147°15′ E	14B26-28	36781-3	
*P. typicus*	*M. fuliginosus*	Hattah Lakes National Park, Vic	34°45′ S, 142°20′ E	DF4	Not applicable	MT396202
*Paramacropostrongylus iugalis*	*M. giganteus*	15 km NW of Nyngan, NSW	31°31′ S, 147°20′ E	14U1	36779-80	MT396203
*P. iugalis*	*M. giganteus*	65 km S of Miles, Qld	26°39′ S, 150°11′ E	49V1	49052	MT396204
*P. iugalis*	*M. giganteus*	5 km south of Reid River, Qld	19°48′ S 146°49′ E	27R1	49048	
*P. iugalis*	*M. giganteus*	Jumba Station via Charters Towers, Qld	21°80′ S, 146°26′ E	50K1	49055	
*P. iugalis*	*M. giganteus*	Melmoth Station via Dingo, Qld	23°25′ S, 149°14′ E	AL12-13	19762	
*P. iugalis*	*M. giganteus*	10 km W of Mungallala, Qld	26°26′ S, 147°31′ E	WW1	49045	
*P. iugalis*	*M. giganteus*	5 km E of Omanama, Qld	28°23′ S, 151°19′ E	49S1	49054	
*P. iugalis*	*M. giganteus*	50 km N of Bourke, NSW	29°33′ S, 145°50′ E	WO6	49047	
*P. iugalis*	*M. giganteus*	Warraweena Station via Bourke, NSW	30°15′ S, 146°07′ E	14H10, 13	36784	
*P. iugalis*	*M. giganteus*	15 km NW of Nyngan, NSW	31°31′ S, 147°20′ E	14U2	36780	
*P. iugalis*	*M. giganteus*	Mullengudgery, NSW	31°42′ S, 147°29′ E	14W2	36788	
*P. iugalis*	*M. giganteus*	Warraweena Station via Bourke, NSW	30°15′ S, 146°07′ E	14H10	36784	
*Paramacropostrongylus toraliformis*	*M. giganteus*	55 km W of Warwick, Qld	28°11′ S, 151°56′ E	49Q1	49053	MT396205
*P. toraliformis*	*M. giganteus*	30 km E of Inglewood	28°24′ S, 151°40′ E	7R6	25688	
*P. toraliformis*	*M. giganteus*	Research, Vic	37°42′ S, 145°11′ E	YD5	49051	MT396206
*P. toraliformis*	*M. giganteus*	Heathcote, Vic	36°54′ S, 144°43′ E	W449	49049	
*P. toraliformis*	*M. giganteus*	St Andrews, Vic	37°35′ S, 145°17′ E	W759	49050	
*P. toraliformis*	*M. giganteus*	10 km N of Bacchus Marsh, Vic	37°37′ S, 144°47′ E	13M10	49042	
*P. toraliformis*	*M. giganteus·*	Lara, Vic	38°00′ S, 144°24′ E	31P6	33088, 34701 49044	
*Hypodontus macropi*	*Wallabia bicolor*	Miles, Qld	26°39′ S, 150°11′ E	RG92/4C21	23985	MT396207(ITS-1)
*H. macropi*	*Notamacropus rufogriseus*	Miles, Qld	26°39′ S, 150°11′ E	XN1	35085	MT396208(ITS-1)

Abbreviations: NSW = New South Wales, Qld = Queensland, SA = South Australia, Vic = Victoria, WA = Western Australia.

**Table 2:** The lengths in base pairs (bp) and GC contents of the unique first (ITS-1) and second (ITS-2) internal transcribed spacer sequences included in the phylogenetic analyses.

Species	Host	Voucher No.	GenBank Accession No.	Length (bp)		GC Content (%)	
				ITS-1	ITS-2	ITS-1	ITS-2
*Hypodontus macropi*	*Notamacropus rufogriseus*	XN1	MT396208	406	292	39.90	38.70
*H. macropi*	*Wallabia bicolor*	RG92	MT396207	417	323	39.80	39.30
*Macropicola ocydromi*	*Macropus fuliginosus*	24C1	MT396193	384	257	42.20	43.20
*Macropostrongyloides baylisi*	*Osphranter r. erubescens*	21P1.1	MK842145	398	251	42.70	40.60
*Macropostrongyloides dissimilis*	*W. bicolor*	10W2	MK842126	392	241	42.80	38.60
*M. dissimilis*	*W. bicolor*	4C14	MK842128	392	237	42.10	38.00
*Macropostrongyloides lasiorhini*	*Lasiorhinus latifrons*	F516	MK842124	385	237	43.10	40.90
*M. lasiorhini*	*Vombatus ursinus*	41R1	MK842123	383	237	43.10	38.40
*Macropostrongyloides mawsonae*	*Macropus giganteus*	41N1.1	MK842146	383	237	43.30	40.50
*Macropostrongyloides spearei*	*Osphranter r. erubescens*	23Q1	MK842135	385	237	43.10	40.90
*Macropostrongyloides woodi*	*Osphranter rufus*	23RQ1.1	MK842135	384	237	43.50	40.90
*Macropostrongyloides yamagutii*	*M. fuliginosus*	14R8	MK842122	383	237	43.10	41.40
*Oesophagostomoides giltneri*	*V. ursinus*	41V1	MT396195	370	217	45.40	40.60
*O. giltneri*	*V. ursinus*	41Z1	MT396194	370	217	45.38	40.60
*Oesophagostomoides longispicularis*	*V. ursinus*	47K.8	MT396196	373	217	45.60	40.10
*Oesophagostomoides stirtoni*	*L. latifrons*	41W1.2	MT396197	372	217	45.20	41.50
*O. stirtoni*	*L. latifrons*	41W1.4	MT396198	372	217	45.20	41.00
*Phascolostrongylus turleyi*	*V. ursinus*	10Z1	MT396200	372	217	45.20	41.50
*Pa. turleyi*	*V. ursinus*	42L 1	MT396199	371	217	46.10	41.90
*Paramacropostrongylus iugalis*	*M. giganteus*	14U1	MT396203	383	241	41.30	39.80
*P. iugalis*	*M. giganteus*	14U2	MT396204	383	241	41.50	39.80
*Paramacropostrongylus toraliformis*	*M. giganteus*	49Q1	MT396205	381	260	42.40	41.50
*P. toraliformis*	*M. giganteus*	YD5	MT396206	381	260	42.30	41.50
*Paramacropostrongylus typicus*	*M. fuliginosus*	DF4	MT396202	383	241	42.00	40.20
*P. typicus*	*M. fuliginosus*	14B28	MT396201	383	241	41.80	40.40

**Table 3:** Pairwise distances (%) within the concatenated first and second internal transcribed spacer (ITS+) among the *Phascolostrongylinae* nematode species included in the phylogenetic analyses.

	*Paramacropostrongylus*						*Phascolostrongylus* and *Oesophagostomoides*							*Macropicola* and *Hypodontus*			*Macropostrongyloides*							
	1	2	3	4	5	6	7	8	9	10	11	12	13	14	15	16	17	18	19	20	21	22	23	24
1. MT396206 *P. toraliformis*																								
2. MT396205 *P. toraliformis*	0.2																							
3. MT396201 *P. typicus*	21.5	21.3																						
4. MT396202 *P. typicus*	21.7	21.5	0.2																					
5. MT396203 *P. iugalis*	21.5	21.3	0.6	0.9																				
6. MT396204 *P. iugalis*	21.6	21.5	0.8	1	0.2																			
7. MT396199 *Pa. turleyi*	22.4	22.3	18	18.3	18	18.2																		
8. MT396200 *Pa. turleyi*	22.4	22.3	18.2	18.4	18.2	18.3	0.2																	
9. MT396195 *O. giltneri*	22.3	22.1	18.3	18.6	18.3	18.5	3.1	2.9																
10. MT396194 *O. giltneri*	22.6	22.4	18.5	18.7	18.5	18.7	3.2	3.1	0.5															
11. MT396197 *O. stirtoni*	22.1	22	18.3	18.6	18.3	18.5	3.9	3.7	2.9	3.1														
12. MT396198 *O. stirtoni*	22.3	22.1	18.2	18.4	18.2	18.3	3.7	3.6	2.7	2.9	0.2													
13. MT396196 *O. longispicularis*	22.4	22.3	18.3	18.6	18.3	18.5	2.5	2.4	1.7	2	3	2.9												
14. MT396193 *M. ocydromi*	22.4	22.2	21.7	21.9	21.9	22	22.5	22.5	22.3	22.5	22.3	22.2	22.1											
15. MT396208 *H. macropi*	28.3	28.5	32.4	32.3	32.4	32.5	33.2	33.2	33.6	33.7	34	33.9	33.8	31.2										
16. MT396207 *H. macropi*	26.7	26.8	28.2	28.1	28.2	28.3	29.4	29.4	28.9	29	29.4	29.3	29.5	26.7	12.3									
17. MK842122 *Ma. yamagutii*	17.8	17.6	19.2	19.4	19	19.2	18.3	18.3	18.6	18.7	18.1	17.9	18.4	18.1	29.4	26								
18. MK842123 *Ma. lasiorhini*	18.7	18.5	20.4	20.6	20.2	20.4	19.2	19.4	20	20.2	19.6	19.4	19.8	19.7	30.3	26.8	4							
19. MK842124 *Ma. lasiorhini*	17.8	17.6	19.4	19.6	19.3	19.4	19	19	19.3	19.5	18.8	18.7	19.1	18	29.7	26.2	3	4.5						
20. MK842127 *Ma. dissimilis*	24.6	24.8	12.2	12.4	12.2	12.3	20.9	21.1	20.9	21.1	21.1	20.9	21.1	24.6	33.2	29.3	20.8	22.3	21.2					
21. MK842128 *Ma. dissimilis*	24.8	24.9	12.4	12.7	12.1	12.3	21.1	21.2	20.8	20.9	20.9	20.8	20.9	24.7	33.5	29.8	20.6	22	21	2.4				
22.MK842130 *Ma. woodi*	19.8	19.7	20.4	20.5	20.2	20.4	20	19.8	20	20.2	19.8	19.7	20	19.2	30.5	27	5.4	5.9	5.6	22.8	22.4			
23. MK842131 *Ma. spearei*	18	17.8	19.6	19.9	19.5	19.6	18.8	18.9	19.2	19.4	19.1	18.9	19.1	18.8	29.3	25.6	2.3	3.8	3	21.1	20.9	5.5		
24. MK842145 *Ma. baylisi*	22.4	22.2	23.4	23.6	23.2	23.4	23.4	23.6	23.7	23.9	23.4	23.3	23.7	21.5	32.8	28.7	11.2	12.4	11	26.3	26.1	11.4	10.9	
25. MK842146 *Ma. mawsonae*	18.7	18.5	19.6	19.8	19.4	19.6	19.6	19.8	19.8	20	19.3	19.2	19.6	19	30.6	27.2	4	5	4	20.7	20.6	6.7	3.6	11.5

## Data Availability

Specimens of worms were deposited in the Australian Helminthological Collection (AHC) of the South Australian Museum, Adelaide (SAM), Australia. The DNA sequences of internal transcribed spacers of the nuclear ribosomal DNA reported in this manuscript are available from the public database, GenBank.

## Supplementary Materials

The following are available online at https://www.mdpi.com/2076-2607/9/1/9/s1, Figure S1: Alignments of the first (A) and second (B) internal transcribed spacers. A dot indicates an identical nucleotide with respect to the top sequence for each alignment. International Union of Pure and Applied Chemistry (IUPAC) codes indicate polymorphic positions in the sequences.
