# Supplementary material for: Phylogenetic Relationships within the Nematode Subfamily Phascolostrongylinae (Nematoda: Strongyloidea) from Australian Macropodid and Vombatid Marsupials
Source: Microorganisms. 2020 Dec 22;9(1):9. doi: 10.3390/microorganisms9010009 (PMC7822168; doi:10.3390/microorganisms9010009)
Supplement: Supplementary file 1 [file microorganisms-09-00009-s001.pdf]

A

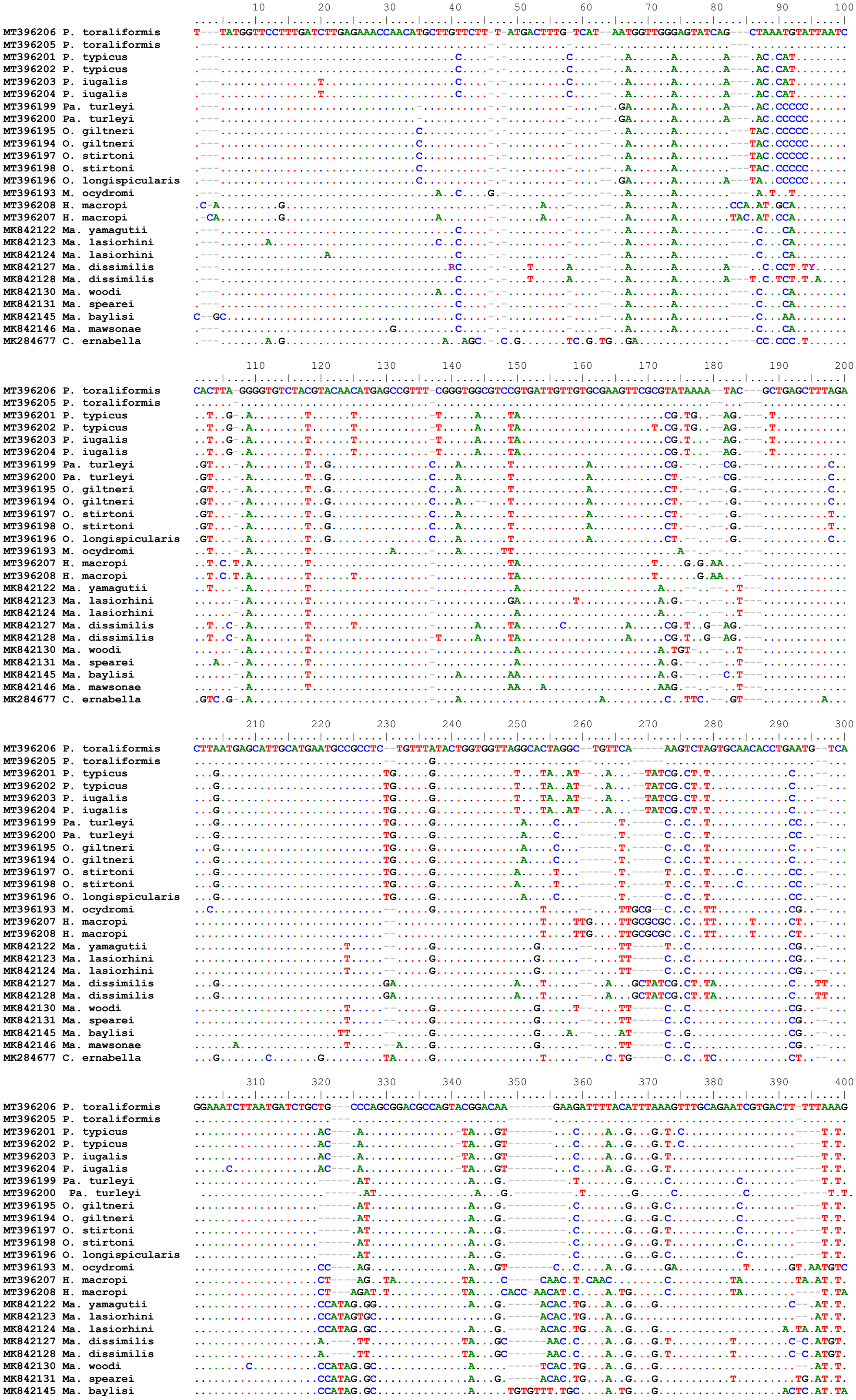

```
MK842146 Ma. mawsonae       ..................CCATAG.GC.............TA...G.-----ACAC.TG...A..G...G....................--.CT.T.
MK284677 C. ernabella       ........C.....CTC...----T.A....AG.......G.A..TG.---------.C.......T.G.G......A...TC.......C----TT..

                                    410       420
                            ....|....|....|....|....|...
MT396206 P. toraliformis     T-------------------CACAATTG
MT396205 P. toraliformis     .-------------------........
MT396201 P. typicus         .-------------------........
MT396202 P. typicus         .-------------------........
MT396203 P. iugalis         .-------------------........
MT396204 P. iugalis         .-------------------........
MT396199 Pa. turleyi        .-------------------......C.
MT396200 Pa. turleyi        .-------------------......C.
MT396194 O. giltneri        .-------------------......C.
MT396197 O. giltneri        .-------------------......C.
MT396196 O. stirtoni        .-------------------......C.
MT396193 O. stirtoni        .-------------------......C.
MT396207 O. longispicularis .-------------------......C.
MT396193 M. ocydromi        A-------------------........
MT396207 H. macropi         .ATATGTATATAAAAAGTCA..A.....
MT396208 H. macropi         .------------AAAGTCA..A.....
MK842122 Ma. yamagutii      .-------------------.....A..
MK842123 Ma. lasiorhini     .-------------------......A.
MK842124 Ma. lasiorhini     .-------------------........
MK842127 Ma. dissimilis     .-------------------........
MK842128 Ma. dissimilis     .-------------------........
MK842130 Ma. woodi          .-------------------........
MK842131 Ma. spearei        .-------------------.....A..
MK842145 Ma. baylisi        .-----------GTCACACA........
MK842146 Ma. mawsonae       .-------------------........
MK284677 C. ernabella       .-------------------......C.
```

B

```
                                     10        20        30        40        50        60        70        80        90       100
                            ....|....|....|....|....|....|....|....|....|....|....|....|....|....|....|....|....|....|....|....|
MT396206 P. toraliformis     TT-ATG-AAT--ACTACAGTGTGGCGTGTAAAG-CACTGTTTGTCGAACGACGCTTGCTTGCCA-TATGTGCGTACGTGCGTTTGTATGTGTATATGGT-
MT396205 P. toraliformis     ..-...-...--.....................-..............................-..................................-
MT396201 P. typicus         .--..AC...--.............C......A-........................CACAT.-...--------------------C.A...G..AA-
MT396202 P. typicus         .--..AC...--.............C.....RA-........................CACAT.-...--------------------C.A...G..AA-
MT396203 P. iugalis         .--..AC...--.............C......A-........................CACAT.-...--------------------C.A...G..AA-
MT396204 P. iugalis         .--..AC...--.............C......A-........................CACAT.-...--------------------C.A...G..AA-
MT396199 Pa. turleyi        .--..A-...--.............T......A-...................T....---A..-.G.--------------------------------
MT396200 Pa. turleyi        .--..A-...--.............T......A-...................T....---A..-.G.--------------------------------
MT396195 O. giltneri        .--..A-...--.............T...G..A-...................T....---A..-.G.--------------------------------
MT396194 O. giltneri        .--..A-...--.............T...G..A-...................T....---A..-.G.--------------------------------
MT396197 O. stirtoni        .--....-..--.............T........-..................T....---A..-.G.--------------------------------
MT396198 O. stirtoni        .--....-..--.............T......A-...................T....---A..-.G.--------------------------------
MT396196 O. longispicularis .--..A-...--.............T...G..AC...................T....---A..-.G.--------------------------------
MT396193 M. ocydromi        .--TCTGC..AC.............C...G..A-........................TACATT.-.C.------------------------C.GGT..-
MT396207 H. macropi         ..C..AT...--........A....T......A-T................T.......GC.T.-...ACATA..T..AT..A.A..CA.A......TGT
MT396208 H. macropi         ..C..AT.TA--........A....T......A-T................T.......GC.T.-...------------ACA.A...A.A.G....TGT
MK842122 Ma. yamagutii      .--.C.A...--.............C....T.A-T................T......---...-...----------------------CC.......-
MK842123 Ma. lasiorhini     .--.C.A...--........A....C....T.A-T................T......---...-...----------------------CC.......-
MK842124 Ma. lasiorhini     .--.C.A...--.............C....T.A-T.......................---...-...----------------------TC.......-
MK842127 Ma. dissimilis     .--..ACT..--.............C....T.A-.......................T---...-C..----------------------TC.....AG-
MK842128 Ma. dissimilis     .--..ACT..--.............C....T.A-.......................T---...-C..----------------------TC.....AG-
MK842130 Ma. woodi          .--.C.A...--.............C....TGA-T............G.A.T...A.---...-...----------------------CC.......-
MK842131 Ma. spearei        .--.C.A...--........A....C....T.A-T..............R.T......---...-...----------------------CC.......-
MK842145 Ma. baylisi        .--.C.A...--.............C....T.A-...............A.T......---T..-...-----------------AT.TCA........G
MK842146 Ma. mawsonae       .--.C.A...--.............C....T.A-..............T..T......---...-...----------------------CC.......-
MK284677 C. ernabella       .--TCAAT..--.............C...T.TA-..............T........T---G..C.GC--------------------------------

                                    110       120       130       140       150       160       170       180       190       200
                            ....|....|....|....|....|....|....|....|....|....|....|....|....|....|....|....|....|....|....|....|
MT396206 P. toraliformis     GCAGTCCTCGTTCTAGGTCAGAA------TTATAT--TGCAACAGGTGCTTT----GG---------AGCA--ATC------------------------
MT396205 P. toraliformis     .......................------......--...............----..---------....--...------------------------
MT396201 P. typicus         ...A........T...A......ACATCTA.....--.......T.......----..---------....--...------------------------
MT396202 P. typicus         ...A........T...A......ACATCTA.....--.......T.......----..---------....--...------------------------
MT396203 P. iugalis         ...A........T...A......ACATCTA.....--.......T.......----..---------....--...------------------------
MT396204 P. iugalis         ...A........T...A......ACATCTA.....--.......T.......----..---------....--...------------------------
MT396199 Pa. turleyi        ..GAC...........A.G....-----TG.G-..--..........A....----..---------..T.--...------------------------
MT396200 Pa. turleyi        ..GAC...........A.G....-----TG.G-..--..........A....----..---------..T.--...------------------------
MT396195 O. giltneri        ..GAC...........A.G....-----TA.G-..--..........A....----..---------..T.--G..------------------------
MT396194 O. giltneri        ..GAC...........A.G....-----TA.G-..--..........A....----..---------..T.--G..------------------------
MT396197 O. stirtoni        ..GAC...........A.G....-----TG.G-..--..........A....----..---------..T.--...------------------------
MT396198 O. stirtoni        ..GAC...........A.G....-----TG.G-..--..........A....----..---------..T.--...------------------------
MT396196 O. longispicularis ..GAC...........A.G....-----TG.G-..--..........A....----..---------..T.--...------------------------
MT396193 M. ocydromi        ...A.........C..A.A....-----T.CT-..--.......T.......----..---------.T...A...------------------------
MT396207 H. macropi         ...A............A.A....-----TAAT-..--.......T.......GTTA..---------G.G.GG...GCTTGTCCACTTATCCGTCCCCCA
MT396208 H. macropi         ...A............A.A....-----TAAT-..--.......T.......GTGT..---------..G.---..GCTTGTCCATTCATCCGTCCCCCA
MK842122 Ma. yamagutii      ...A....T....A..A.A....-----T.C.-.C--...............----..---------..T.--...------------------------
MK842123 Ma. lasiorhini     .T.A....T....A..A......-----T...-.C--...............----..---------..T.--..T------------------------
MK842124 Ma. lasiorhini     ...A....T....A..A......-----T.C.-.C--...............----..---------..T.--...------------------------
MK842127 Ma. dissimilis     ...A............A......ACATCTC....AA........T.......----A.---------..T.--...------------------------
MK842128 Ma. dissimilis     .A.A............A......ACATCTA....A--.......T......C----A.---------..T.--...------------------------
MK842130 Ma. woodi          .T.A....T....A..A......-----TAC.-.C--...............----..---------..T.--...------------------------
MK842131 Ma. spearei        ...A....T....A..A......-----T.C.-.C--...............----..---------..T.--...------------------------
MK842145 Ma. baylisi        ...A....T....A..A...A..A----T.C.-.C--............T..----..---------..TGTTC..------------------------
MK842146 Ma. mawsonae       ...A....T....A..A......-----T.C.-.C--...............----..---------..T.--...------------------------
MK284677 C. ernabella       A..A.....A......A.G....------C.G-.C--C......T..A.A..----..---------...T--TG.------------------------

                                    210       220       230       240       250       260       270       280       290       300
                            ....|....|....|....|....|....|....|....|....|....|....|....|....|....|....|....|....|....|....|....|
MT396206 P. toraliformis     -CAT--------------TGCACA-CGAATGTCATG------------ACAA------ACCTAGTTGTCACTGTGAGTTGCATTTAGCGAA--TAAA-AA
MT396205 P. toraliformis     -...--------------......-...........------------....------.................................--....-..
MT396201 P. typicus         -...--------------......-.AG........------------.AC.----------...........C..AG............C--..TG-..
MT396202 P. typicus         -...--------------......-.AG........------------.AC.----------...........C..AG............C--..TG-..
MT396203 P. iugalis         -...--------------......-.AG........------------.AC.----------...........C..AG............C--..TG-..
MT396204 P. iugalis         -...--------------......-.AG........------------.AC.----------...........C..AG............C--..TG-..
MT396199 Pa. turleyi        -...--------------..T...-...........------------..CT----------C.............AA-.....C.....C--...G-..
MT396200 Pa. turleyi        -...--------------..T...-...........------------..CT----------C.............AA-.....C.....C--...G-..
MT396195 O. giltneri        -T.A--------------..T...-...........------------..CT-----------.............AA-...........C--....-..
MT396194 O. giltneri        -T.A--------------..T...-...........------------..CT----------C.............AA-...........C--....-..
MT396197 O. stirtoni        -..A--------------..T...-...........------------..CT----------C.............AA-...........C--....-..
MT396198 O. stirtoni        -..A--------------..T...-...........------------..CT----------C.............AA-...........C--....-..
MT396196 O. longispicularis -..A--------------..T...-...........------------.TCT-----------.............AA-...........C--....-..
MT396193 M. ocydromi        -..C----------ATTGC.................-------------TG.CACACATAA.C.............AA............C--AT..-..
MT396207 H. macropi         T...--------------..T...-..........AAGACACACACAC...CACACAC.TA.C.....T.......AA............GAA.TGG-..
MT396208 H. macropi         T...--------------..T...-..........A------------...T----AC.TA.C.....T.......AA............GAA.TG.-..
MK842122 Ma. yamagutii      -...--------------......-...........------------.T.C--CACT..AAC.............AG............C--....-..
MK842123 Ma. lasiorhini     -...--------------......-..........C------------.T.C--CATA..AAC.............AG............T--....-..
MK842124 Ma. lasiorhini     -...--------------......-...........------------.T.C--CACA.TA.C.............AG............C--....-.G
MK842127 Ma. dissimilis     -T..--------------......-..G.....TG.------------.TC.--------TG......T.....A.AG............CTT.TTG-..
MK842128 Ma. dissimilis     -T..--------------......-..G.....TG.------------.TC.--------TG......T.....A.AG............C--.TTG-..
```

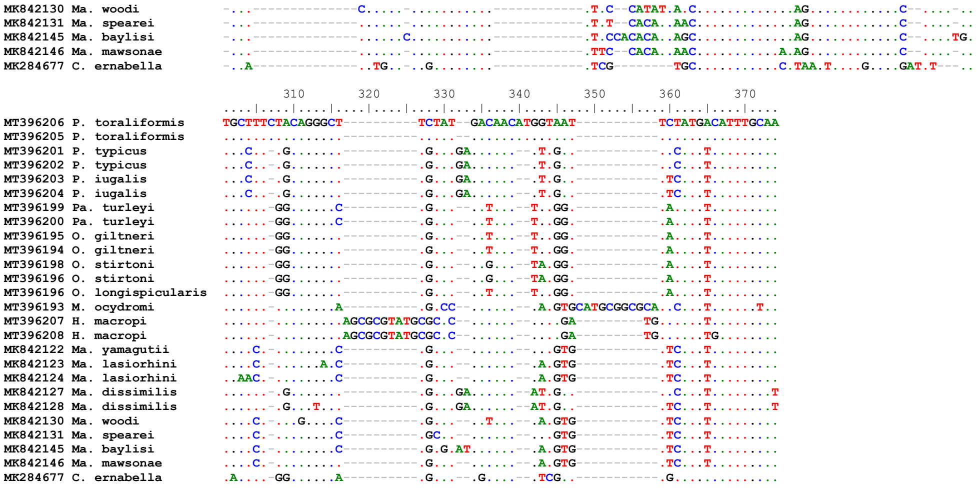

Figure S1. Alignments of the first (A) and second (B) internal transcribed spacers. A dot indicates an identical nucleotide with respect to the top sequence for each alignment. International Union of Pure and Applied Chemistry (IUPAC) codes indicate polymorphic positions in the sequences.
